# Diagnostic Value of Liquid-Based Cytology in Urothelial Carcinoma Diagnosis: A Systematic Review and Meta-Analysis

**DOI:** 10.1371/journal.pone.0134940

**Published:** 2015-08-04

**Authors:** You Luo, Dong-Li She, Hu Xiong, Li Yang, Sheng-Jun Fu

**Affiliations:** Gansu Nephro-Urological Clinical Center, Institute of Urology, Department of Urology, Key Laboratory of Urological Disease of Gansu Province, Lanzhou University Second Hospital, Lanzhou 730000, China; Centro Nacional de Investigaciones Oncológicas (CNIO), SPAIN

## Abstract

**Objective:**

To evaluate the value of liquid-based cytology (LBC) in the diagnosis of urothelial carcinoma.

**Method:**

Diagnostic studies were searched for the diagnostic value of LBC in urothelial carcinoma in PubMed, Embase, Cochrane Library, Web of Science, CBM and CNKI. The latest retrieval date was September 2014. The data were extracted and the quality of the included studies was independently assessed by 2 reviewers. Stata 13 software was used to perform the statistical analysis. The research was conducted in compliance with the PRISMA statement.

**Result:**

Nineteen studies, which included 8293 patients, were evaluated. The results of the meta-analysis showed that the pooled sensitivity and specificity of LBC were 0.58 (0.51–0.65) and 0.96 (0.93–0.98), respectively. The diagnostic odds ratio (DOR) was 31 (18–56) and the area under the curve (AUC) of summary receiver operating characteristic (SROC) was 0.83 (0.80–0.86). The post-test probability was 80% when a positive diagnosis was made. Compared with high grade urothelial carcinoma (HGUC), the sensitivity of detecting low-grade urothelial carcinoma (LGUC) was significantly lower, risk ratio of sensitivity was 0.54 (0.43–0.66), P<0.001. However, no significant sensitivity improvement was observed with LBC when compared with traditional cytospin cytology, risk ratio was 1.03 (0.94–1.14), P = 0.524.

**Conclusion:**

Despite LBC having a pooled 58% positive rate for urothelial carcinoma diagnosis in our meta-analysis, no significant improvement in sensitivity was observed based on the studies evaluated. Further research is needed to validate these findings.

## Introduction

Urothelial carcinoma (UC) is one of the most common urologic cancers. Among urogenital tumors, it is only second to prostate cancer. The majority of UCs is bladder cancer, and the incidence of bladder cancer ranked 11^th^ in global cancer statistics and seventh in malignancy for male tumors [1]. In clinical practice, cystoscopy with a biopsy is the gold standard for diagnosis. It’s aggressive and relatively inconvenient as a follow-up monitoring approach. Urinary cytology is a noninvasive examination for urothelial carcinoma but has a low and unstable detection rate. The sensitivity and specificity of urinary cytology are 13–75% and 85–100%, respectively. Additionally, urinary cytology is likely to be influenced by external factors [2,3]. Liquid-based cytology (LBC) is widely used in cervical cancer screening but has been confined to this application since it was approved by the FDA in 1996. Recently, this slide-making technique has been noted in other medical fields, such as breast cancer, sputum cytology of lung cancer, and especially urinary cytology. Some studies indicate that LBC may improve the sensitivity of bladder cancer detection and the background of micrographs [4–7]. We evaluated the diagnostic value of the LBC techniques in urothelial carcinoma with evidence-based medicine methods.

## Materials and Methods

### Search strategy

A literature search of PubMed, Embase, Web of Science, Cochrane Library, Chinese BioMedical Literature Database (CBM) and Chinese National Knowledge Infrastructure (CNKI) was conducted by two independent investigators (DLS and YL) to retrieve the clinical researches through to September 2014. The search terms used were “liquid based cytology”, “thinprep”, “autocyte”, “surepath”, “cellpreplus”, “thin layer”, “monolayer”, “bladder cancer” and “urothelial carcinoma”. References in the retrieved literature and previous systematic reviews were identified for any relevant studies. The eligibility criteria in this study included any cytological diagnostic study including the LBC techniques. Studies with the following criteria were excluded: (1) the research subjects were special patients or had unusual situations, such as confinement to atypical cells or low-to-high grade urothelial carcinoma; (2) related data could not be extracted nor calculated; or (3) duplicate published articles or articles with overlapping patients. This systematic review was performed in compliance with the Preferred Reporting Items for Systematic Reviews and Meta-Analyses statement (PRISMA) [8].

### Quality assessment and data extraction

The quality of the included studies was independently assessed by QUADAS-2 [9], which was recommended for a diagnostic accuracy review by Cochrane Systematic Review [10]. The data extraction was performed and cross-checked by 2 independent investigators (YL and DLS). If there was a discrepancy in opinions, it was solved by group discussion. The information extracted from these citations included the name of the first author, year of publication, regions, urine sample, et al. The data were extracted from the original outpatient data, if possible. The extracted data included the number of true positives (tp), false positives (fp), false negatives (fn) and true negatives (tn) using LBC and traditional cytospin, if the data were available. The data from patients with previous urothelial carcinoma were not excluded. When a study included no direct data, we calculated the incidence using a basic formula (such as “sensitivity” = tp/(tp+fn), “specificity” = tn/(tn+fp)).

### Statistical analysis

Stata 13 (Stata Corp LP, College Station, TX, USA) was used to conduct the synthesis analysis and perform the publication bias detection. The effect size included sensitivity, specificity, positive likelihood ratio, negative likelihood ratio, diagnostic odds ratio, area under the curve (AUC) and post-test probability, and bivariate mixed-effects binary regression model was used [11]. When compared sensitivity of different cytology techniques and different tumor grades, Cochrane’s Q test and *I* ^*2*^ statistic were performed for heterogeneity. P≥0.1 and *I* ^*2*^ <50% indicated there was no significant heterogeneity, in which case a fixed model (Mantel-Haenszel method) was applied. Otherwise was significant heterogeneity, a random model (Der Simonian and Laird method) was used. A comparison of LBC and traditional cytospin cytology was also performed, and the risk ratio (RR) was measured. Leave-one-out method was used for the sensitivity analysis. Publication bias for diagnostic accuracy test was detected by Deek’s funnel plot asymmetry test [12]. A P≤0.10 was considered significant publication bias. A 2-tailed P≤0.05 indicated statistical significance for other than outcomes of publication bias test.

## Results

### Literature filtration and quality assessment

**Fig 1:**
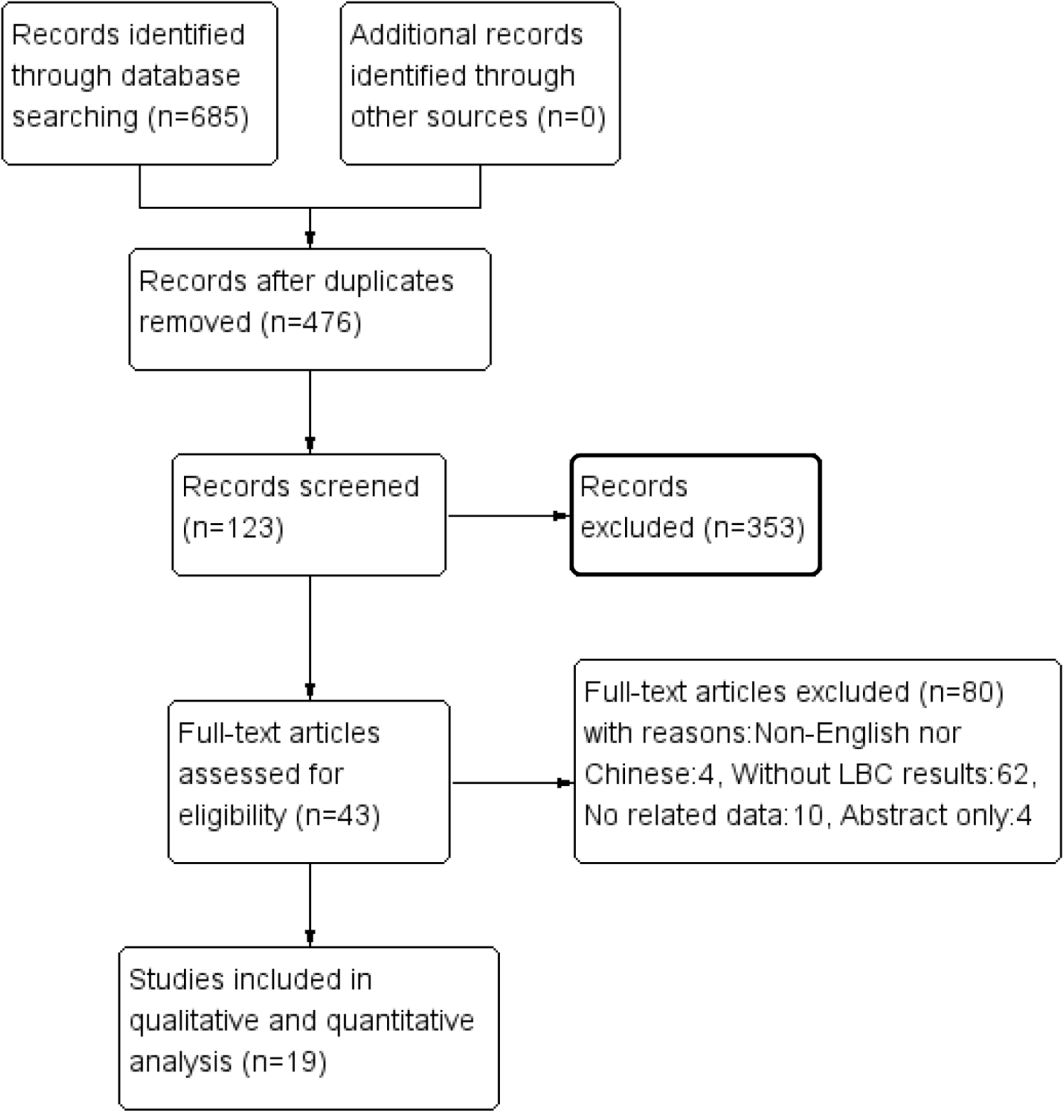
Flow diagram of studies retrieved.

**Fig 2:**
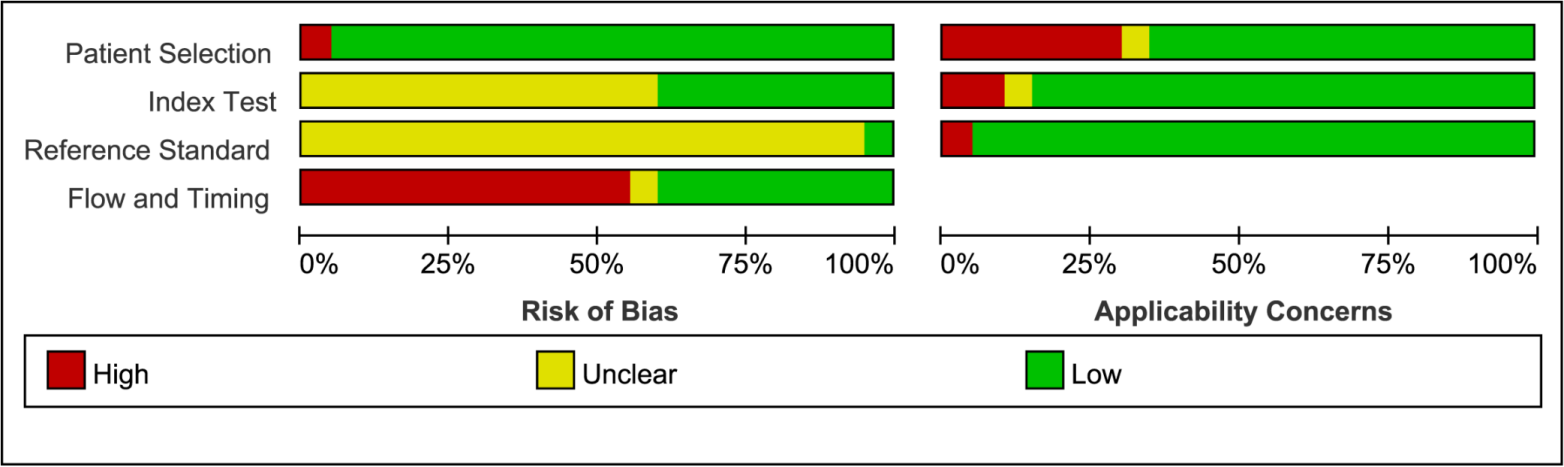
Quality assessment of the included studies.

Six hundred eighty-five citations were searched using the initial search strategy. No meta-analyses had been published. Nineteen citations [13–31], which included 15 English references [13,15–24,26–29] and 4 Chinese articles [14,25,30,31], were included. No randomized controlled trials were included. The 19 studies included 8293 patients (1933 cancer patients and 6360 control patients). The screening flow chart is shown in Fig 1, and Table 1 shows the basic characteristics of the included studies. The quality assessment of included studies is shown in Fig 2 as a comprehensive result determined by QUADAS-2.

**Table 1:** Characteristics of included studies.

Studies [reference]	Year	Regions	Gold Standard	Cancer type	Cytology diagnostic category	Stain	Urine Samples	tp	fp	fn	tn
Lodde [19]	2003	Italy+Austria	His	uc	N\A\S\P	Pap+HE	vu	22	2	29	38
Lodde [20]	2004	Italy+Austria	Cys+Bio	bc	N\A\S\P	Pap+HE	vu	21	5	26	49
Piaton [22]	2005	France	Cys+His	buc	N\A\S\P	Pap	vu+cu	42	4	19	133
Lodde [18]	2006	Italy+Austria	Cys+bio	buc	N\A\S\P	Pap	vu	49	9	52	167
Mian [21]	2006	Italy	Cys+His	buc	N\A\S\P	Pap	vu	116	10	182	1578
Hwang [15]	2007	Korea	Bio	bc	N\A\S\P	Pap	NA	18	1	11	43
Sng [26]	2007	Singapore	Bio	uc	N\A\P	Pap	vu	16	1	4	5
Zhao [31]	2008	China	His	uc	N\A\S\P	Pap	vu	188	13	86	83
Sullivan [28]	2009	USA	Cys+Bio	bc	N\A\P	Pap	vu	5	2	19	72
Ye [30]	2011	China	Cys+Bio	bc	N\A\S\P	HE	vu	39	1	21	147
Saeb-Parsy-Aberdeen [24]	2012	UK	Cys+Bio	bc	N\A-S\P	Pap	vu	15	11	6	85
Saeb-Parsy-Cambridge [24]	2012	UK	Cys+Bio	bc	N\A-S\P	Pap	vu	10	12	6	38
Son [27]	2012	Korea	His	bc	N\A\S\P	Pap	vu	25	0	26	37
Li [17]	2013	China	His	uc	N\A\S\P	Pap	vu	76	6	28	55
Shen [25]	2013	China	Cys+His	buc	NA	NA	vu	28	0	32	62
van Hemel [29]	2013	Netherlands	His	uc	Benign\Abnormal	Pap	NA	61	17	57	40
Dimashkieh [13]	2013	USA	Cys+His	bc	N\A\S\P	NA	vu+wu+cu	15	11	30	734
Piaton [23]	2014	France	Cys+His	uc	N\A-US\A-H\P	Pap	NA	85	5	21	50
Li [14]	2014	China	Cys+His	bc	N\A\S\P	HE	vu	50	1	18	127
Kim [16]	2014	Korea	TURB+Bio	bc	N\A\S\P	Pap	wu	231	143	148	2563

Abbreviations: uc-urothelial carcinoma, bc-bladder cancer; vu-void urine, wu-washing urine, cu-catheterized urine; Cys-cystoscopy, His-histology, Bio-biopsy; N-negative, A-atypical, S-suspicious, P-positive, A-US—atypical unknown significance, A-H—atypical cannot exclude high grade; Pap-papanicolaou, HE-hematoxylin eosin; NA-not applicable; tp-true positive, fp-false positive, fn-false negative, tn-true negative.

### Diagnostic results

**Fig 3:**
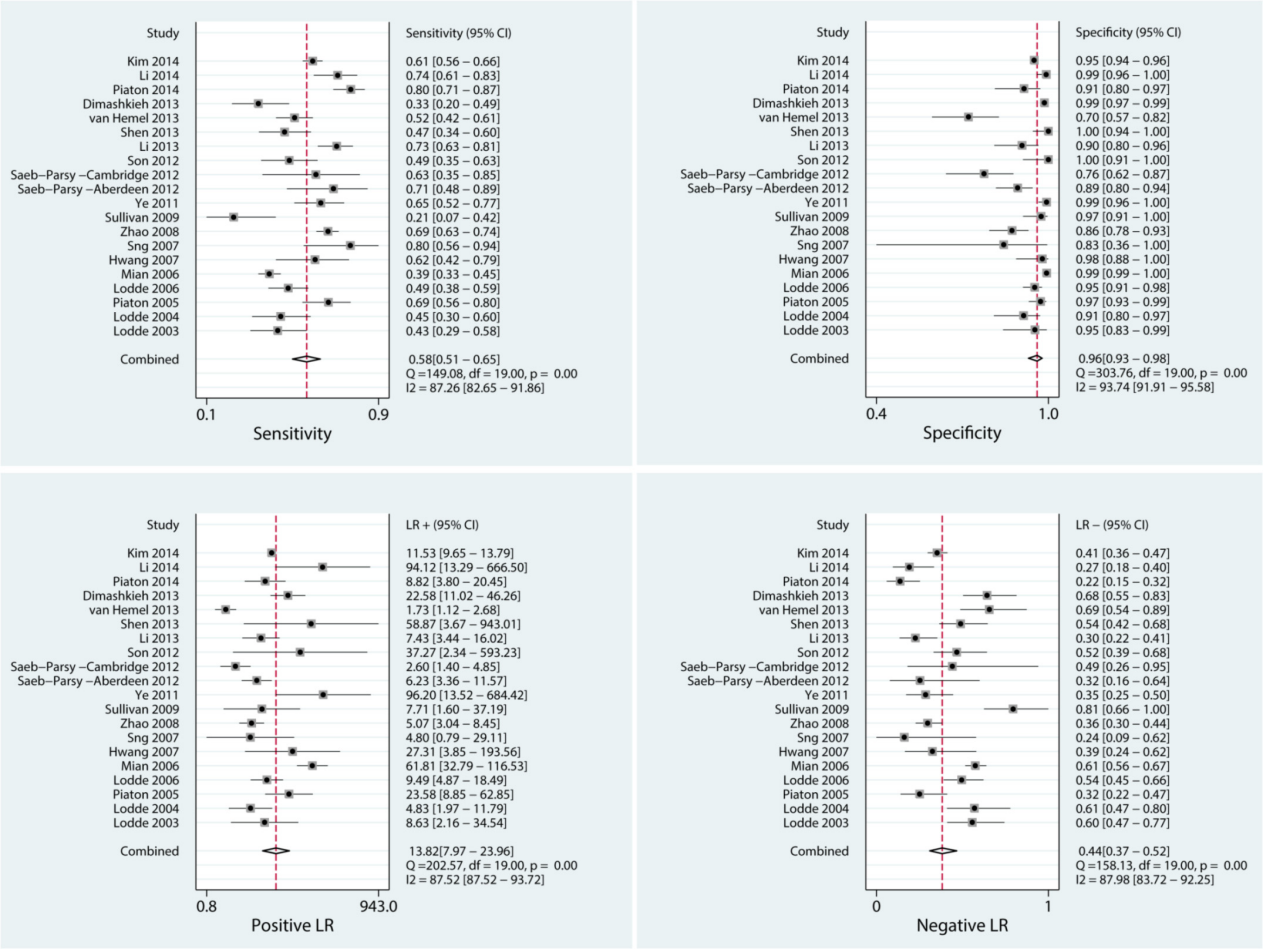
Pooled sensitivity, specificity, positive DLR and negative DLR.

**Fig 4:**
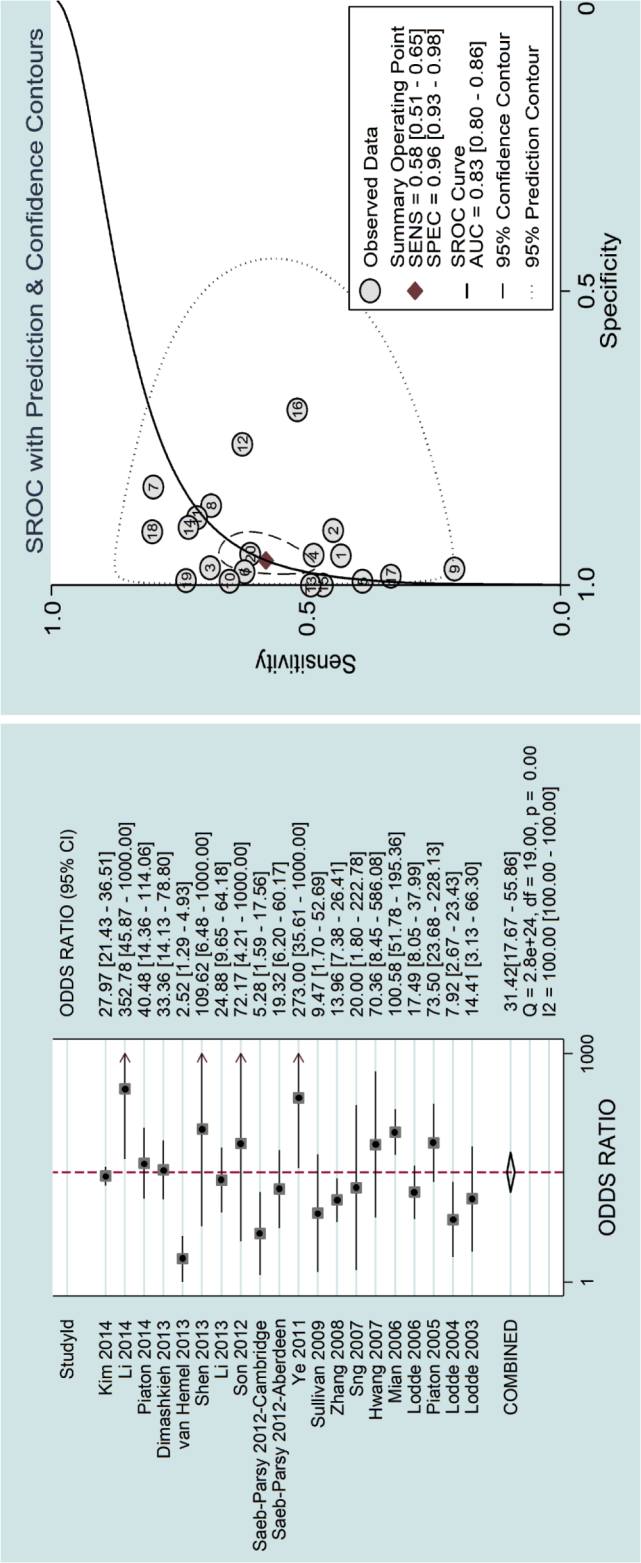
Pooled diagnostic odds ratio and SROC of the included studies.

Before the quantitative analysis, we conducted a threshold effect, the result of which was a Spearman correlation coefficient of 0.361, P = 0.118>0.10, which could be considered no significant threshold effect. Then, the quantitative analysis yielded the comprehensive results shown in Figs 3 and 4, The pooled sensitivity and specificity of LBC were 0.58 (0.51, 0.65) and 0.96 (0.93, 0.98), respectively. The pooled positive and negative likelihood ratios were 13.8 (8.0, 24.0) and 0.44 (0.37, 0.52), respectively. The pooled DOR was 31 (18, 56). The pre-test probability was 0.23, and the positive and negative post-test probability was 80% and 12%, respectively. The AUC of the summary receiver operating characteristic (SROC) was 0.83 (0.80, 0.86), which indicated a moderate diagnostic value. All pooled results were tabulated in Table 2.

**Table 2:** Pooled diagnostic results of included studies.

Effect Size	Sensitivity	Specificity	LR-	LR+	DOR
Pooled Results	0.58 (0.51–0.65)	0.96 (0.93–0.98)	0.44 (0.37–0.52)	13.82 (7.97–23.96)	31.42 (17.67–55.86)
P value of Q test	<0.01	<0.01	<0.01	<0.01	<0.01
I^2^ statistic	87.26%	93.74%	87.98%	87.52%	100%

Abbreviations: LR-: negative likelihood ratio. LR+: positive likelihood ratio. DOR: diagnostic odds ratio.

#### LBC versus CS

**Fig 5:**
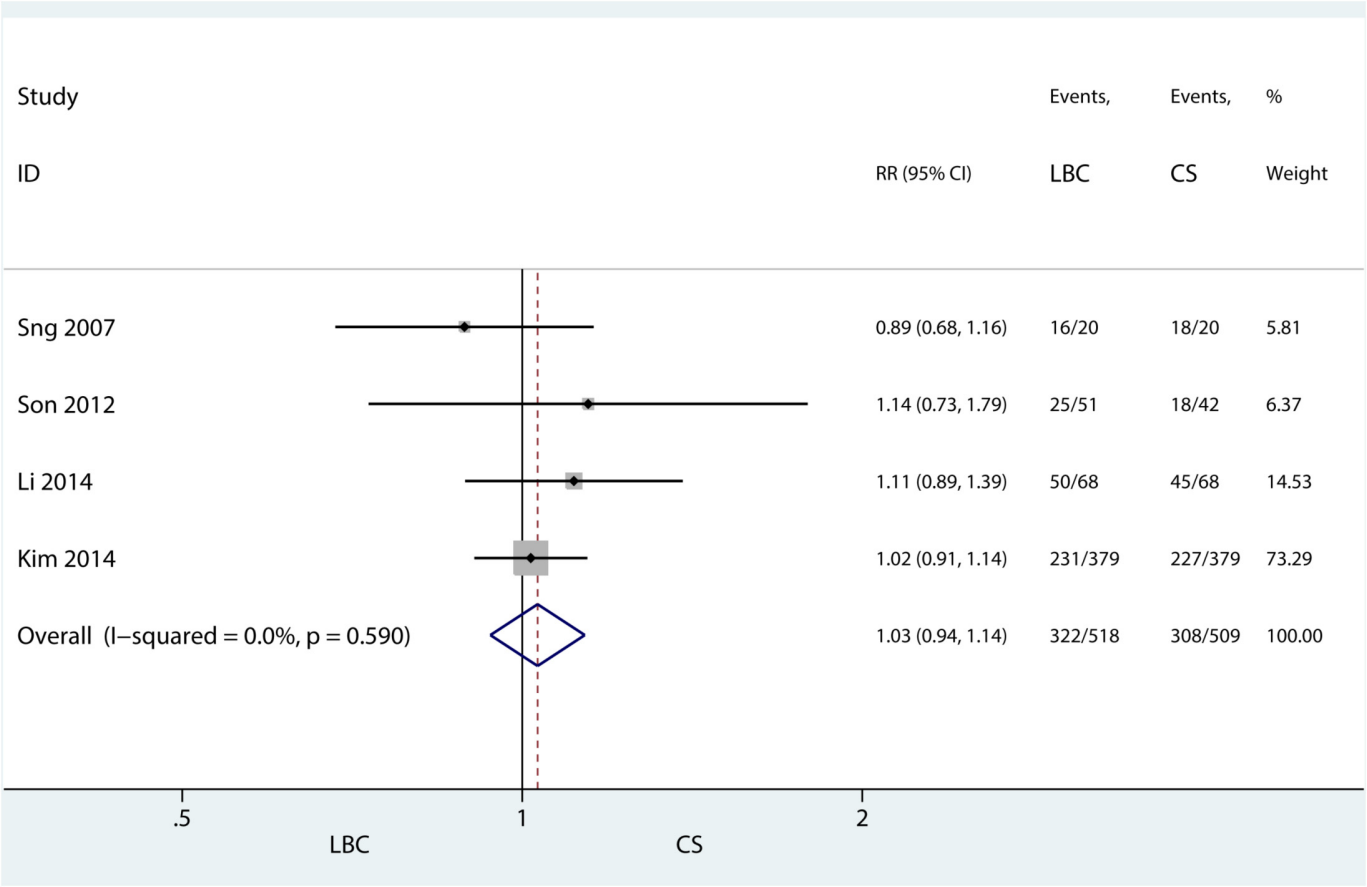
Comparison of sensitivity of Liquid Based Cytology versus Traditional Cytospin Cytology.

**Fig 6:**
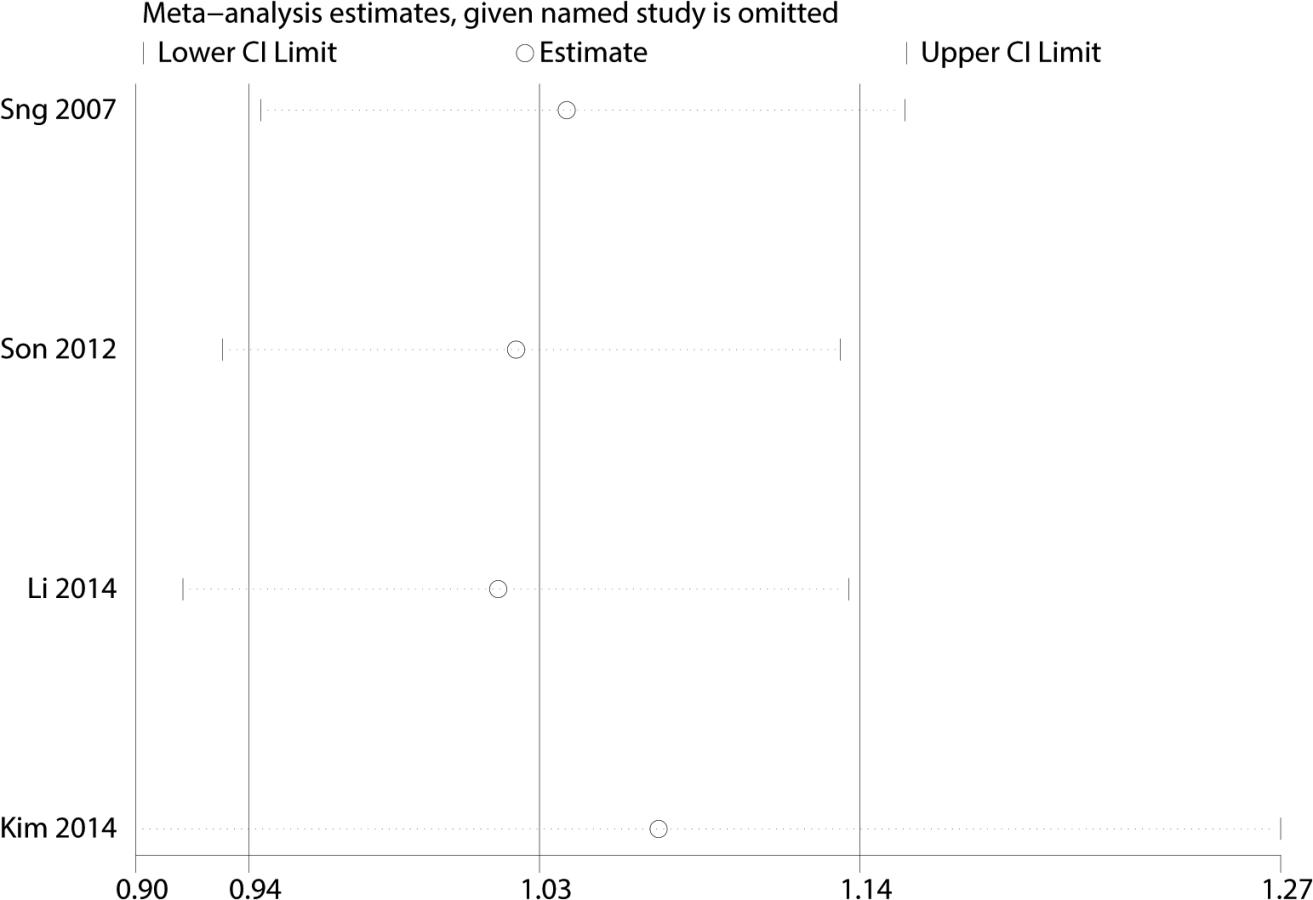
Sensitivity analysis of Comparison of sensitivity of LBC versus CS.

Only 4 studies [14,16,26,27] (Fig 5) of the 19 citations compared the sensitivity of LBC and traditional cytospin cytology (CS). I^2^ and P value were 0% and 0.590, respectively. Fixed model was used and the synthetic risk ratio (RR) was 1.03 (95%CI 0.94–1.14), P = 0.524, which indicated that LBC did not yield a significant improvement in the sensitivity of detection. Sensitivity analysis did not alter the outcome (Fig 6).

#### Tumor grade category

**Fig 7:**
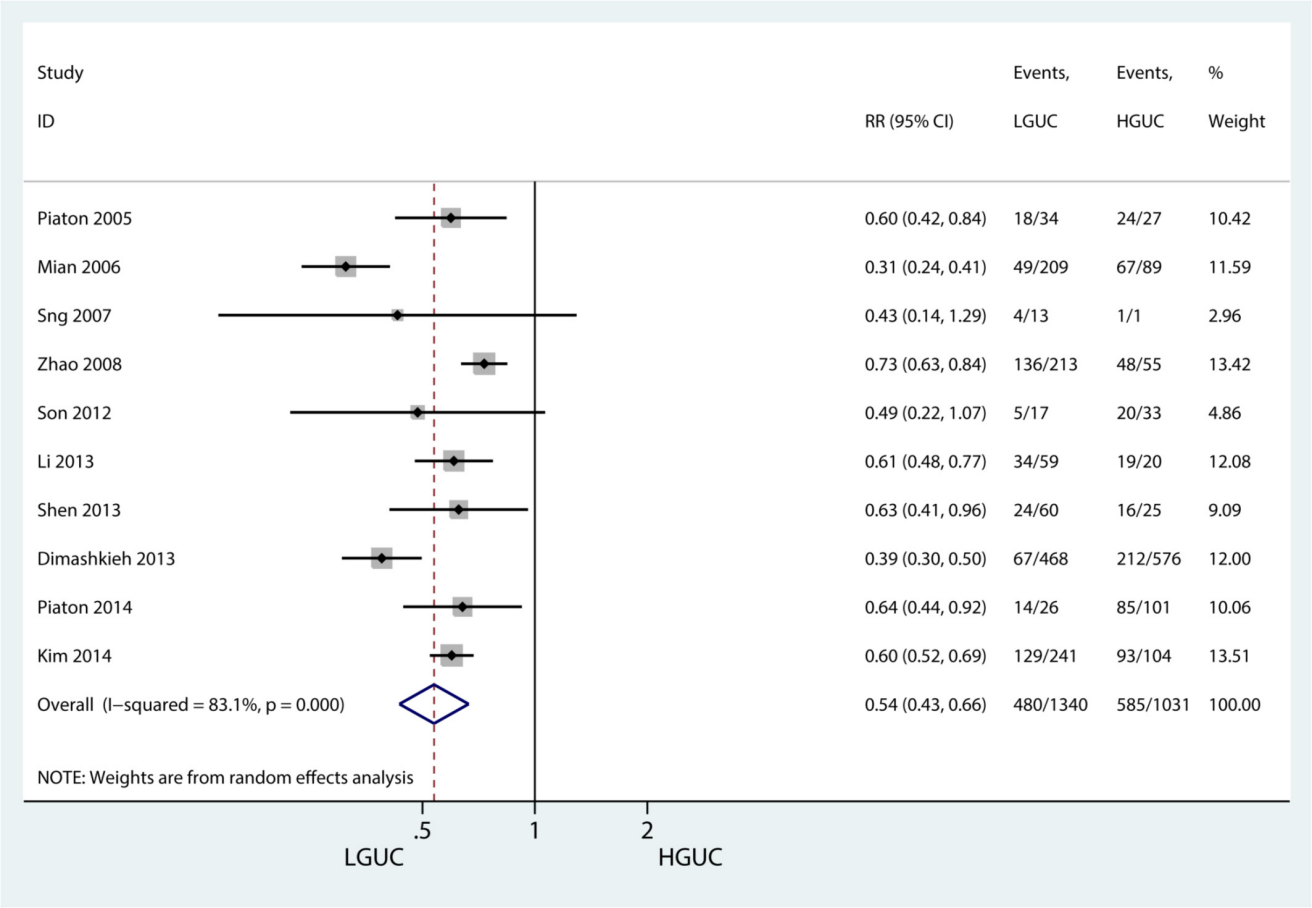
Sensitivity of LGUC versus HGUC.

A sensitivity comparison was performed between the low-grade and high-grade urothelial carcinoma, in which G1 and G2 were set as the low-grade urothelial carcinoma (LGUC) and G3 as the high-grade urothelial carcinoma (HGUC). Both I^2^ of 83.1% and the P value of Cochrane Q test showed significant heterogeneity. Random effect model was used. The forest plot showed that LGUC cytological diagnosis had a significantly lower sensitivity than HGUC, RR = 0.54 (0.43, 0.66), P<0.001 (Fig 7). Sensitivity analysis did not change the significance (The result was omitted).

#### Publication bias

**Fig 8:**
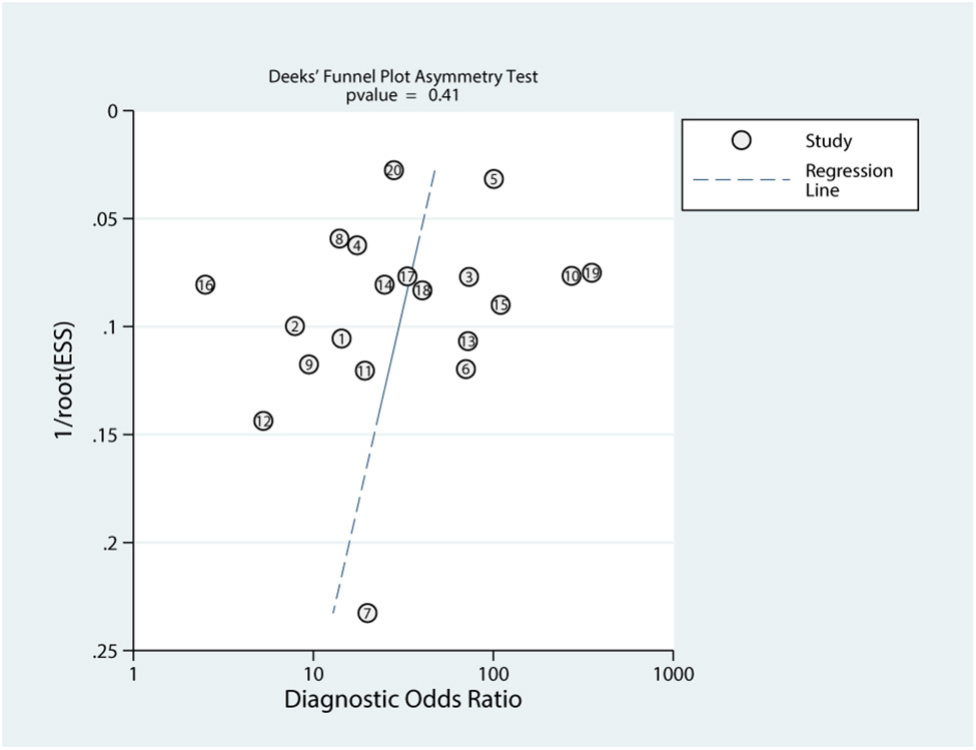
Publication bias of included studies. 1/root(ESS) meant inverse root of effective sample sizes. Each circle represented an included study.

The result of Deek’s plot is shown in Fig 8 which indicates absence of significant publication bias (P = 0.41).

## Discussion

Urinary cytology has a long history of widespread use and irreplaceable clinical detection. As a noninvasive exam, urinary cytology had the disadvantage of relatively low and unstable sensitivity, which has led to its limited application. Additionally, a traditional urinary cytology cytospin slide only shows a few cells, which can be confounded with red blood cells, white blood cells and other non-urothelial cells or impurities. This could decrease the identification of urothelial cells. LBC is an innovative slide-making technique that was initially applied in cervical cancer screening, and it greatly reduced the slide-making time, obliterated the non-urothelial cells and mucus in the urine, humidified slides and decreased cell degeneration by a preservation solution, and improved slide quality (including background, cell dispersion, and reducing the confounding cells) [5–7,32]. Additionally, the automatic processing of LBC is another positive feature [27]. Above all, LBC reduces the probability of atypical urothelial cells on the slide, which causes low and unstable cytological sensitivity [6,7].

Many studies have verified that the sensitivity of urinary cytology differed between the urothelial carcinoma grades: the higher grade, the higher sensitivity [33,34]. This may result from well-differentiated low-grade urothelial carcinoma causing indistinguishable cell morphology and a tight connection between cancer cells with a minimal probability to fall off. Our pooled study with LBC confirmed these results. Compared with HGUC, LGUC detection had a significantly lower sensitivity, RR = 0.54 (0.43, 0.66) P<0.001. The different urine acquisition approaches were another impact factor affecting the sensitivity. Wash urine or bladder irrigation was more sensitive than catheterized. Void urine had the lowest sensitivity [35,36]. Few studies compared the methods using urine category; thus, the urine category subgroup was not analyzed. Additionally, another decisive impact factor was staining, which determined the observational method and results. The HE stain is a traditional staining method for urinary cytology, while for the LBC stain, the majority were stained with papanicolaou (Pap). Because the principal focus of cytology for urothelial carcinoma is the cell nucleus, both HE and Pap stained the cell nucleus. Acridine orange fluorescence improved sensitivity to 77.1% and was associated with tumor grade and stage. Higher sensitivity was observed with acridine orange fluorescence than the Feulgen stain, and the lowest sensitivity was observed with the HE stain [37]. However, combining LBC with different staining methods to improve sensitivity of urinary cytology has yet to be verified by further studies.

Currently, research hotspots regarding urothelial carcinoma detection are concentrated on tumor markers, such as NMP22, BTA, and Survivin [38]. A noticeable improvement in sensitivity was shown when LBC was used for uCyt+/immunoCyt in bladder cancer test [19,21,39], but this test had low specificity, and was more expensive and time consuming with lower efficiency. The current trend is to combine LBC with other laboratory tests for urothelial carcinoma detection.

The following are the strengths of our meta-analysis: This is a first meta-analysis that evaluates the diagnostic accuracy of liquid based cytology in urothelial carcinoma. Additionally, we compared the sensitivity between liquid based cytology and traditional cytospin cytology and presented no sensitivity improvement was observed. Additionally, the fact that LBC versus CS pooled effect yielded an I-square value of 0% and the sensitivity analysis showed a stable pooled effect. Meanwhile, our meta-analysis has some limitations. First, some studies on LBC diagnostic accuracy of urothelial carcinoma in outpatients were limited in cured patients with urothelial carcinoma, which led to a selection bias of patients. Second, blinding in most of the included studies was not certain. Third, a comparison between LBC and traditional cytospin cytology was conducted in only a few studies. Fourth, the heterogeneity could not be avoided. Most other forest plots were heterogeneous (high I-square values) which are limitations. The cytology diagnostic category, stain methods and urine samples possibly affect the heterogeneity of the included studies. Hence, higher quality diagnostic trials comparing LBC with cytospin cytology are expected to confirm our results.

## Conclusion

In summary, our study showed comprehensive diagnostic results for LBC including sensitivity, specificity, positive and negative likelihood ratios, diagnostic odds ratio. Low-grade urothelial carcinoma diagnosis had a significantly lower sensitivity than high-grade urothelial carcinoma. Based on the few included references comparing LBC with traditional cytospin cytology, LBC did not show any improvement in sensitivity. More studies are needed for further validation. However, the automation and excellent slides prepared using the LBC techniques made the slide-making process highly efficient, which could a good reason to promote this technique.

## Supporting Information

S1 Checklist PRISMA 2009 Checklist. (DOC)
